# Amyotrophic lateral sclerosis and cerebellum

**DOI:** 10.1038/s41598-022-16772-5

**Published:** 2022-07-22

**Authors:** Renata Kabiljo, Alfredo Iacoangeli, Ammar Al-Chalabi, Ivana Rosenzweig

**Affiliations:** 1 Department of Neuroimaging, Sleep and Brain Plasticity Centre, Institute of Psychiatry, Psychology and Neuroscience (IoPPN), King’s College London, London, UK; 2 Department of Basic and Clinical Neuroscience, Maurice Wohl Clinical Neuroscience Institute, Institute of Psychiatry, Psychology and Neuroscience, King’s College London, London, SE5 9NU UK; 3 Department of Biostatistics and Health Informatics, Social, Genetic and Developmental Psychiatry Centre, Institute of Psychiatry, Psychology and Neuroscience, King’s College London, De Crespigny Park, Denmark Hill, London, SE5 8AF UK

**Keywords:** Amyotrophic lateral sclerosis, Computational biology and bioinformatics

## Abstract

Amyotrophic lateral sclerosis (ALS) is a devastating, heterogeneous neurodegenerative neuromuscular disease that leads to a fatal outcome within 2–5 years, and yet, a precise nature of the association between its major phenotypes and the cerebellar role in ALS pathology remains unknown. Recently, repeat expansions in several genes in which variants appreciably contribute to cerebellar pathology, including *C9orf72*, *NIPA1*, *ATXN2* and *ATXN1*, have been found to confer a significant risk for ALS. To better define this relationship, we performed MAGMA gene-based analysis and tissue enrichment analysis using genome-wide association study summary statistics based on a study of 27,205 people with ALS and 110,881 controls. Our preliminary results imply a striking cerebellar tissue specificity and further support increasing calls for re-evaluation of the cerebellar role in the ALS pathology.

## Introduction

Amyotrophic lateral sclerosis (ALS) is a devastating, heterogeneous neurodegenerative neuromuscular disease predominantly affecting upper and lower motor neurons^1^, leading to death within 2–5 years^1^. About 15% of people with ALS have mutations in one of the 40 Mendelian ALS genes^1^. Recently, repeat expansions in several genes in which variants appreciably contribute to cerebellar pathology, including *C9orf72*, *NIPA1*, *ATXN2* and *ATXN1*, have been found to confer a significant risk for ALS^1,2^.

Cerebellar degeneration in ALS has long been a contentious topic, with the consensus being minimal involvement of the cerebellum in ALS, or at best, a compensatory role for cerebellar function during progressive supratentorial degeneration^3,4^. This is, however, in opposition to compelling radiological and post-mortem pathologic evidence for extrapyramidal and cerebellar degeneration^4–6^. Accordingly, a recent imaging study of 161 people with ALS, stratified for ALS-associated *C9orf72* and *ATXN2* variants, described distinct focal cerebellar trophic change, preferentially affecting specific lobules^5^. Notably, a significant cerebellar pathology was also demonstrated in patients without these ALS-associated mutations^5^.

Based on these findings^2,5^, we explored whether significant cerebellar specificity of the ALS phenotypes could be confirmed by performing MAGMA tissue expression analyses on the ALS genome-wide association study (GWAS) summary statistics.

## Results

The ten most statistically significant genes in MAGMA gene-based analysis were *MOB3B, C9orf72* (unless excluded), *SCFD1, UNC13A, IFNK, G2E3, TNIP1, TBK1, BAG6* and *EFTUD1*. Complete list and MAGMA-dataset is available from https://fuma.ctglab.nl/browse/423.

Of 54 anatomical regions investigated, MAGMA-tissue-expression-profile-analysis revealed that the ALS-associated genes were significantly enriched for expression in the cerebellum and the cerebral-cortex [*P*(*cerebellum*) = 1.3 × 10^−04^; *P*(*cerebellar_hemispheres*) = 1.5 × 10^−04^; *P*(*brain_frontal_cortex_BA9*) = 3.3 × 10^−04^ and *P*(*brain_cortex*) = 1.2 × 10^−04^].

**Figure 1:**
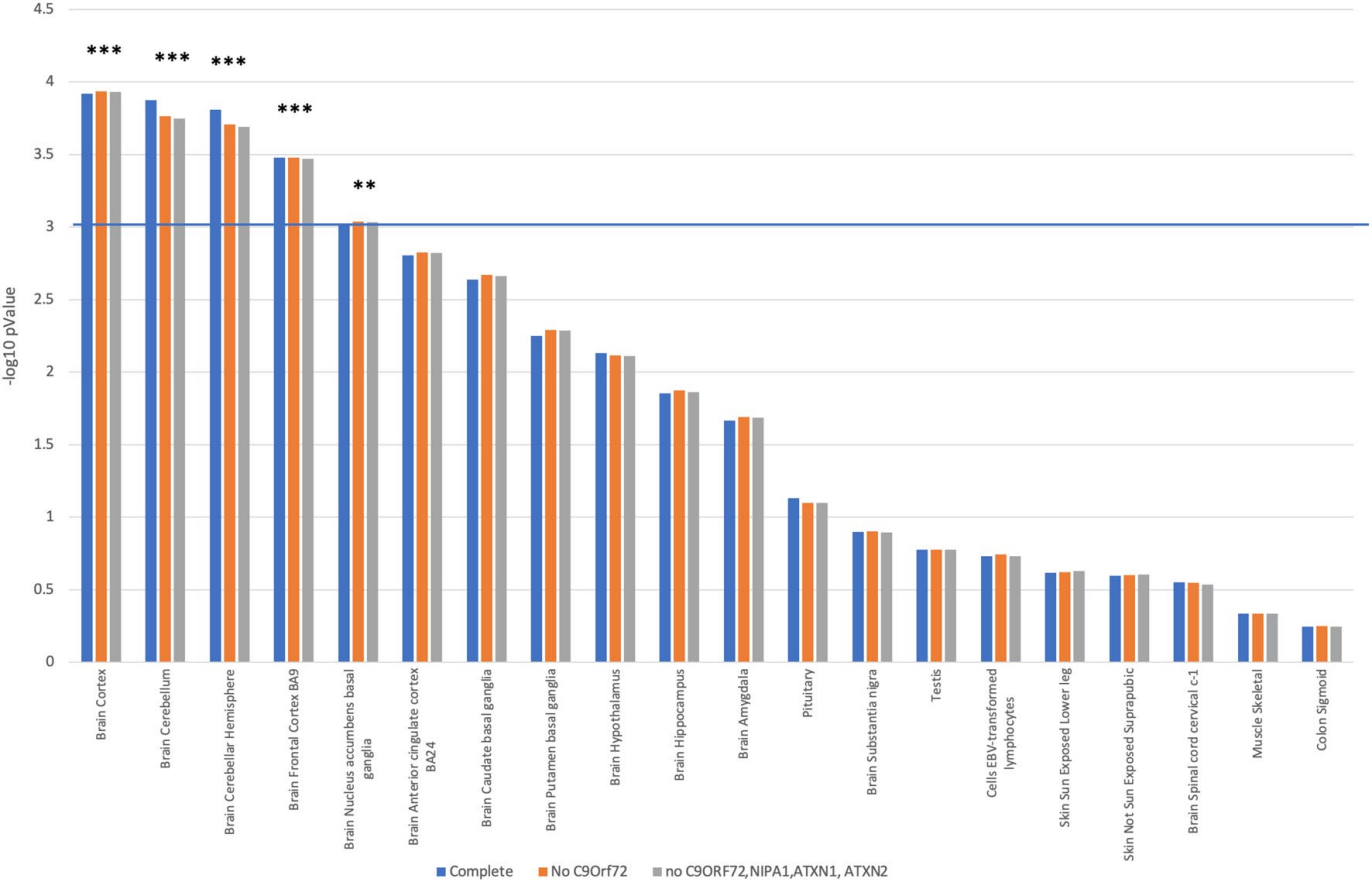
MAGMA tissue enrichment analysis of candidate genes for ALS, based on GTEx RNA-seq data of the 54 specific tissue types. Top 20 tissues are shown in figure. Significant tissues are marked with *.

This enrichment was observed even when known cerebellar pathology-associated ALS-risk genes *C9orf72*, *ATXN1*, *ATXN2* and *NIPA1* were excluded in later analyses to avoid disproportionate enrichment (Fig. 1). It is of note that the region of the nucleus accumbens within the basal ganglia reached statistical significance during these analyses [*P(nucleus accumbens)* = 9.2 × 10^−04^]. Statistical significance for the most enriched tissues is listed in Table 1.

**Table 1:** *P* values for the most enriched tissues for MAGMA tissue enrichment analysis of candidate genes for ALS, based on GTEx RNA-seq data for 54 specific tissue types.

Anatomical region	Complete	No *C9ORF72*	No *C9ORF72,NIPA1,ATXN1, ATXN2*
Brain cortex	**0.00012057**	**0.00011576**	**0.00011708**
Brain cerebellum	**0.00013406**	**0.00017257**	**0.00017868**
Brain cerebellar hemisphere	**0.00015471**	**0.00019659**	**0.00020407**
Brain frontal cortex BA9	**0.00033164**	**0.00033169**	**0.00033791**
Brain nucleus accumbens basal ganglia	0.00098116	**0.00092179**	**0.00092735**
Brain anterior cingulate cortex BA24	0.0015641	0.0015002	0.0015101
Brain caudate basal ganglia	0.0023117	0.0021419	0.0021732
Brain putamen basal ganglia	0.0056439	0.0051024	0.005195
Brain hypothalamus	0.0073602	0.0076545	0.007772
Brain hippocampus	0.01405	0.01342	0.013692
Brain amygdala	0.021646	0.020465	0.020641
Pituitary	0.074125	0.079439	0.080076
Brain substantia nigra	0.12635	0.12497	0.12759
Testis	0.16764	0.16718	0.16765
Cells EBV-transformed lymphocytes	0.18536	0.18119	0.18576

Significant *P* values are bolded.

## Discussion

We report a striking cerebellar tissue specificity for ALS. In addition, similar specificity is shown for the dorsolateral-prefrontal-region (the-Broadmann-area-9), the cortical-area targeted with distinct cerebellar inputs via thalamic-projections, essential for ‘higher’-cognitive functions such as working-memory, motor-planning, abstract reasoning and voluntary control of automatic movements^7^.

Moreover, we report that this specificity remains even when we exclude ALS-genetic variants known to contribute to cerebellar pathology in ALS.

In past, ALS has been similarly associated with widespread and differential basal ganglia involvement^8^. More specifically, changes in the regions of the nucleus caudatus, hippocampus, and in the region of the nucleus accumbens, have been proposed to present some of the key features of ALS^8^. Accordingly, these brain regions feature among the top ten enriched anatomical regions (see Fig. 1, Table 1). Statistically significant specificity has, however, only been demonstrated for the basal ganglia’s nucleus accumbens region (Table 1), and only in analyses that excluded the ALS-genetic variants known to contribute to cerebellar pathology in ALS, including *C9orf72*. This is perhaps somewhat contraintuitive to previous studies, which argued a more intensive basal ganglia involvement in patients with ALS carrying the *C9orf72* hexanucleotide repeat expansion^8^. Whilst the dissonance may reflect important aspects of our methodological limitations, we propose that our findings may also emphasise the complexity of the cerebellar role in the ALS-affected neurocircuitry. For example, a specific (focal) cerebellar pathology may arguably dictate differential downstream changes in functional connectivity between the sub-regions of the cerebellum, the dorsolateral prefrontal cortex and the nucleus accumbens^9,10^. The cerebellum shares functionality in motivated behaviors^9,10^ with these subcortical and cortical regions, and thus, any distinct cerebellar changes may drive and underlie, at least in part, different ALS phenotypes, with significant clinical implications^9^.

In summary, the role of the cerebellum in exacerbating cardinal clinical manifestations such as motor disability, bulbar dysfunction, respiratory compromise, sleep and cognitive problems, is often overlooked, and symptoms traditionally primarily linked to supratentorial pathology^4^. Furthermore, a closed-loop connectivity between localised regions of the prefrontal cortex, nucleus accumbens and cerebellum, and the extent to which cerebellar output may contribute to the ALS pathology remain mostly unmapped. Further aggravating point is that it is also challenging to identify cerebellar signs clinically in patients with motor weakness.

Our findings cannot be taken to suggest causality, or indeed the valence of these cerebellar associations due to the methodological limitations of MAGMA-analyses. Nonetheless, while cerebellar signatures of specific ALS-genotypes are yet to be firmly established, our study further supports increasing calls for re-evaluation of the cerebellar role in the ALS pathology^4,5^.

## Methods

For the purpose of this study, MAGMA^11^ gene-based analysis and tissue enrichment analysis were performed using genome-wide association study (GWAS) summary statistics from a study of 27,205 people with ALS and 110,881 controls^12^, downloaded from https://surfdrive.surf.nl/files/index.php/s/E5RetKw10hC3jXy.

Three MAGMA-analyses were performed. During the first we analysed the entire GWAS-ALS dataset. To establish whether genes with known cerebellar involvement might be driving potential enrichment in cerebellum, we performed two additional analyses. For the first, all SNPs mapping positionally to *C9orf72* were excluded (see Table 1, column ‘No *C9orf72’*. To the same end, additionally, all SNPs mapping positionally to *C9orf72*, *ATXN1*, *ATXN2* and *NIPA1* were excluded (see Table 1, the column ‘No *C9orf72, ATXN1, ATXN2 and NIPA1*’). MAGMA (v1.08) was invoked by FUMA (v1.3.7)^13^, an online tool for mapping and annotation of genetic associations. In MAGMA gene-based analysis, GWAS summary statistics are used to compute gene-based *P* values for protein coding genes by mapping SNPs to genes if SNPs are located within the genes. Bonferroni correction was used to correct for multiple testing.

Tissue-enrichment analysis was performed using the results of the gene-based analysis and the data from the Genotype—Tissue Expression (GTEx) project^14^, integrated in FUMA (v1.3.7)^13^. GTEx project traditionally includes 54 specific human body tissue types, amongst which are thirteen different brain regions^14^. Detailed information on the anatomical sampling sites, used databanks and the specific extraction methods can be found on https://www.gtexportal.org/. For example, for the cerebellar hemisphere please refer to https://www.gtexportal.org/home/tissue/Brain_Cerebellar_Hemisphere and for the cerebellum on https://www.gtexportal.org/home/tissue/Brain_Cerebellum.

Average gene-expression per tissue type was used as a gene covariate to test for a positive relationship between gene expression in a specific tissue type and genetic associations.

### Ethics declarations

This study does not does report on experiments on humans. Only GWAS summary statistics have been used.

## Data Availability

The complete FUMA gene based and tissue besed analysis results and parameters are available at https://fuma.ctglab.nl/browse/423.
